# A narrative review on the use of autologous platelet concentrates during alveolar bone augmentation: Horizontal (simultaneous/staged) & vertical (simultaneous/staged)

**DOI:** 10.1111/prd.12604

**Published:** 2024-08-28

**Authors:** J. Blanco, J. Caramês, M. Quirynen

**Affiliations:** ^1^ Department of Surgery and Medical‐Surgical Specialties (Area of Stomatology. Unit of Periodontology), Faculty of Medicine and Odontology University of Santiago de Compostela Santiago de Compostela Spain; ^2^ Department of Oral Surgery and Implantology, Unit of Oral Surgery, Faculty of Dental Medicine University of Lisbon Lisbon Portugal; ^3^ Department of Oral Health Sciences, KU Leuven & Dentistry (Periodontology) University Hospitals Leuven Leuven Belgium

**Keywords:** APCs, bone regeneration, horizontal augmentation, simultaneous approach, staged approach, vertical augmentation

## Abstract

This review aimed to answer the general question of whether autologous platelet concentrates (APCs, an autologous blood‐derivative) can improve the outcome of alveolar bone augmentation. Three clinical scenarios were assessed: horizontal/vertical bone augmentation in combination with implant placement (simultaneous approach), horizontal bone augmentation in a staged approach, and vertical bone augmentation in a staged approach. An electronic literature search strategy was conducted for each review from the outset to July 1st, 2023. The titles and abstracts (when available) of all identified studies were screened and imported into a database. If articles appeared to meet the inclusion criteria or their title and abstract had insufficient data, the full text was obtained to make the final decision. All studies that met the inclusion criteria underwent data extraction. Moreover, the references of the identified papers were screened for additional studies. After title and abstract screening and selection criteria application, 14 clinical studies were included for the qualitative analysis: seven for horizontal/vertical bone augmentation in a simultaneous approach, five for horizontal bone augmentation in a staged approach, and two for vertical bone augmentation in a staged approach. There is scarce literature regarding the added value of APCs in bone augmentation, and most studies had small sample sizes, a lack of standardized protocols, and different outcome variables, which makes comparisons between studies difficult. Out of the 14 studies, four were well‐designed randomized clinical trials, where we could find better results for the APCs groups. Most studies, particularly comparative and well‐designed studies, demonstrated beneficial and promising results of using APCs in alveolar bone augmentation. However, before high‐level evidence‐based conclusions can be drawn, more randomized clinical trials must compare the benefits of adding APCs to the gold‐standard approach.

## 1 INTRODUCTION

Alveolar bone resorption after tooth loss can lead to an insufficient bone volume, which might negatively affect the prognosis of dental implants,^1^ even though the fate of the buccal bone is still uncertain. Numerous techniques, such as guided bone regeneration (GBR), bone blocks, and distraction osteogenesis, have been described to reconstruct deficient alveolar ridges. Several materials may be used in those procedures, including autografts, allografts, xenografts, and alloplasts, as well as different barrier membranes or osteosynthesis materials. Thus, in some situations, bone augmentation procedures can be performed simultaneously with implant insertion, whereas in others, the reconstructed ridge requires a healing period, resulting in a delayed, non‐simultaneous implant placement.^2^, ^3^, ^4^

Horizontal bone augmentation has yielded highly predictable results.^5^, ^6^ In the simultaneous approach (simultaneous augmentation and implant insertion), GBR is associated with superior outcomes compared to other procedures and has become the treatment of choice^7^, ^8^ with optimal results in the long‐term.^9^ Moreover, whenever the biological principle of GBR was followed, regeneration occurred irrespective of the type of biomaterial used.^10^ However, regardless of the biomaterial employed, a certain resorption of the augmented bone was observed overtime.^10^, ^11^ Thus, a composite bone graft combining a xenograft with particulate autologous bone has been proposed to increase the osteogenic properties of the graft.^12^, ^13^ In the “staged” approach, autologous bone blocks are the most frequently used technique.^7^ Unfortunately, this technique shows an increased morbidity (due to the need for a second surgical site) and more postoperative complications. Furthermore, varying degrees of graft resorption during the healing phase have been reported.^7^, ^14^, ^15^

Vertical ridge augmentation (in both simultaneous and staged approaches) has been reported as successful but with a lower degree of predictability and a rather higher complication rate than the horizontal approach.^4^, ^16^, ^17^, ^18^, ^19^ The GBR technique seems to achieve greater bone gain than bone blocks and Ti‐meshes in the staged approach and with less superficial resorption.^20^, ^21^ Moreover, the GBR technique appears to show fewer complications (healing and surgical) than others for vertical bone augmentation.^20^, ^22^

Tissue engineering has been applied to further improve bone regeneration. Successful tissue engineering relies on two fundamental principles: a space‐maintaining scaffold and a matrix that permits cell recruitment and neovascularization, besides delivering morphogenetic, regulatory, and growth factors.^23^ Various substances have been tested to promote bone formation, including bone morphogenetic protein (BMP), platelet‐derived growth factor, and transforming growth factor, which are abundant within platelets.^24^ Over the past decades, autologous blood derivatives have been used for soft and hard tissue regeneration. Autologous platelet concentrates (APCs) are among the most used and represent a category of bioactive substances derived from the patient's own blood through a chairside centrifugation process. Four main types of APCs have been employed and can be divided into two broad classifications based on their physical characteristics and content: platelet‐rich plasma (PRP) types, representing liquid platelet suspensions that can be transformed into a fibrin gel after activation; and platelet‐rich fibrin (PRF) types, which form solid fibrin clots because of a highly polymerized three‐dimensional (3D) fibrin network (for details see Calciolari et al.^10^; Blanco et al.^25^; Gruber et al.^26^; Quirynen et al.^27^).

In this context, a question remains unanswered: can the use of APCs during a bone grafting procedure improve bone regeneration or eventually streamline the surgery?

### 1.1 Classification of bony defects

**FIGURE 1:**
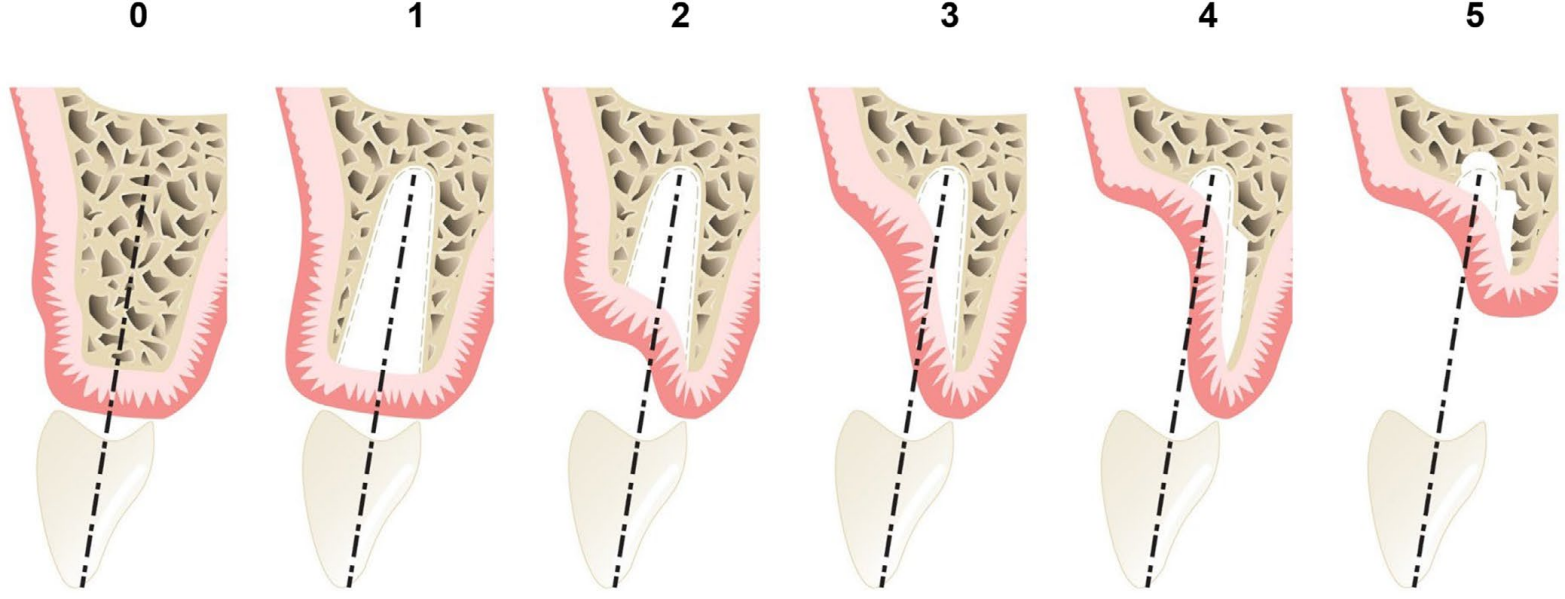
Classification of bony defects.^14^ Class 0: Bony envelope in which an implant can be placed in a prosthetically correct position but where a bone augmentation procedure is indicated to improve the contour of the ridge. Class 1: A gap between the implant surface and intact surrounding bone walls (often observed during immediate implant placement). Class 2: Bony defect restricted to the buccal/lingual aspect but with intact mesial/distal bony conditions/walls. Class 3: Bony dehiscence in which the adjacent bone walls do not provide the volume stability of the area to be augmented. CIass 4: Reduced ridge width precluding the primary stability of the implant in the prosthetically correct position, so a staged approach for bone regeneration is required. Class 5: Similar to Class 4, but also includes vertical ridge resorption.

Different surgical approaches are required depending on the anatomy of the bony defect. This paper will follow the bony defect classification introduced by Benic and Hämmerle^14^ (Figure 1).

### 1.2 Terminology

#### 1.2.1 APC categories

APCs are divided into four families based on their leukocyte content and fibrin structure, as detailed in the literature (Calciolari et al.^28^ Dohan et al.^29^, ^30^; Pietruszka et al.^31^; Varshney et al.^32^):

- Pure platelet‐rich plasma (P‐PRP): without leukocytes and with a low‐density fibrin network (e.g., plasma rich in growth factors (PRGF));
- Leukocyte‐ and platelet‐rich plasma (L‐PRP): contains leukocytes (the amount depends on the protocol) and a low‐density fibrin network (most other PRPs);
- Pure platelet‐rich fibrin (P‐PRF): without leukocytes and with a high‐density fibrin network (infrequently used);
- Leukocyte‐ and platelet‐rich fibrin (L‐PRF): with leukocytes (the amount depends on the protocol) and a high‐density fibrin network or an injectable, flowable subcategory.

#### 1.2.2 Sticky bone

In 2010, Sohn introduced the term “sticky bone” for the first time at a meeting in Tokyo.^33^ Sticky bone is a mixture of autologous fibrin glue and a bone graft that provides stability to the bone graft in the defect, thus accelerating tissue healing and minimizing graft resorption during the healing period. Autologous fibrin glue is obtained by centrifuging venous blood at 2.400–2.700 rpm for 2 min, which results in two layers: a deeper layer of red blood cells and a superficial layer of autologous fibrin glue (plasma including white blood cells, platelets, and fibrinogen). It can also be prepared using various centrifugation protocols with only plasma (PRP, PRGF, or PRF). The resultant sticky bone is moldable, prevents micro and macro movement of grafted bone, entraps platelets and leukocytes in its fibrin network, is natural, and prevents ingrowth of soft tissues in the graft.^34^ It also releases some growth factors.^35^

#### 1.2.3 L‐PRF bone block

In 2018, Cortellini et al. introduced an “upgraded” sticky bone: the L‐PRF bone block, which combines pieces of chopped L‐PRF membranes and liquid fibrinogen (both prepared chairside from the patient's blood) with a particulate bone substitute. The bone substitute embedded in a fibrin matrix (due to the conversion of fibrinogen into a 3D fibrin network) provides a slow resorbing scaffold, and the chopped L‐PRF membrane pieces create extra space between the graft particles, allowing cell ingrowth and neovascularization from the surrounding tissue and facilitating new bone formation. The L‐PRF pieces contain activated platelets secreting a wide range of bioactive molecules and growth factors playing a key role in bone healing and regeneration (for details, see Barthold et al.^36^; Calciolari et al.^28^; Blanco et al.^25^). In an in‐vitro study, Castro et al.^37^ observed that an L‐PRF bone block releases these growth factors for more than 7 days.

**FIGURE 2:**
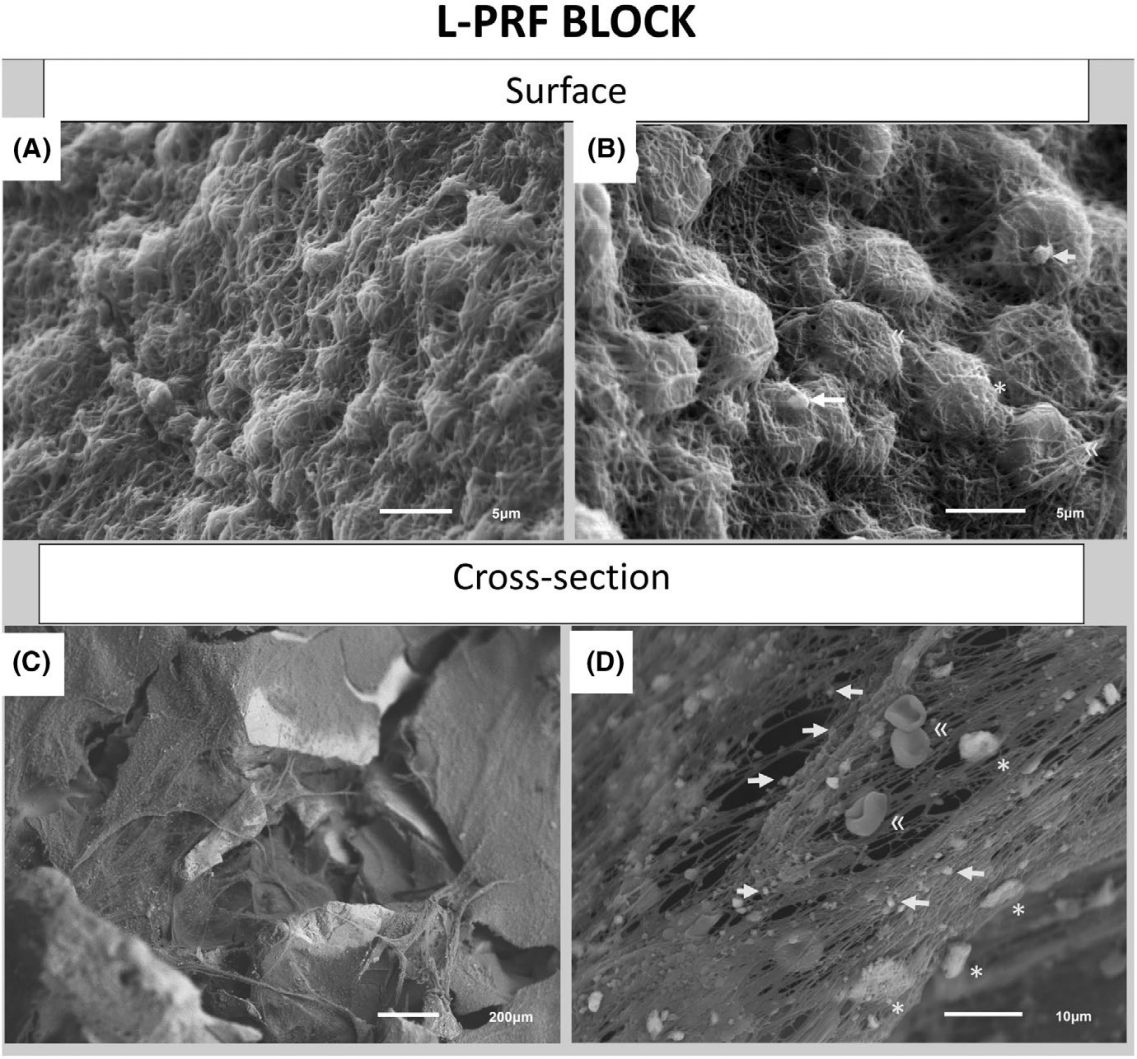
Scanning electron microscope (SEM) images of leukocyte‐ and platelet‐rich plasma (L‐PRF) bone block (surface and cross‐section). (A) Dense fibrin network with embedded cells covering the block; (B) Cells embedded in the fibrin network; (C) Fibrin fibers connecting the Deproteinized Bovine Bone Mineral (DBBM) particles inside the block; (D) Cells in the fibrin network at higher magnification (→: Platelets, *: Leucocytes, and «: Red blood cells),^37^ with enough space for cell migration and angiogenesis.

Liquid fibrinogen connects the bone substitute and L‐PRF pieces (Figure 2). The liquid fibrinogen coagulation starts immediately when it comes in contact with the chopped L‐PRF membranes and is completed within ≤1–2 min, trapping the biomaterial into a block. This block has a form‐proof consistency and light elasticity that allows it to adapt to the recipient site. It can be shaped into the desired form during the first minute.

Scarano et al.^38^ compared the mechanical properties of bovine bone mixed with autologous platelet liquid, blood, or physiologic water. They observed a significant increase in compressive resistance when applying the platelet liquid (18 N for platelet liquid, 10 N for blood, and 2 N for physiological water). Good graft stability is, of course, crucial for the regeneration process. Moreover, the fibrin interconnectivity within the graft diminishes soft‐tissue and epithelium ingrowth.^33^

## 2 MATERIALS AND METHODS

This section is divided into three different parts according to the clinical scenario:

- Horizontal/vertical bone augmentation in combination with implant placement: simultaneous approach;
- Horizontal bone augmentation with the staged approach; and
- Vertical bone augmentation with the staged approach.

Three narrative reviews with a systematic search strategy were performed to assess whether APCs can improve the outcome of alveolar bone augmentation in the three clinical scenarios.

### 2.1 Search strategy

An electronic literature search strategy was conducted for the three reviews from the outset to July 1st, 2023.

#### 2.1.1 Horizontal/vertical bone augmentation with the simultaneous approach

A primary search was performed on MEDLINE using the following combination of search terms: [“osseointegration” OR “dental implants, single‐tooth” OR “dental implants” OR “tooth implant” OR “guided bone regeneration” OR “bone regeneration” OR “alveolar ridge augmentation” OR “bone augmentation” OR “alveolar bone loss” OR “bone resorption”] AND [“platelet‐rich fibrin” OR “autologous platelet concentrate” OR “thrombocyte rich plasma” OR “leukocyte platelet‐rich fibrin” OR “pure platelet‐rich fibrin” OR “LPRF” OR “L‐PRF” OR “advanced platelet‐rich fibrin” OR “APRF” OR “A‐PRF” OR “CGF” OR “L‐PRF Gel”]. Only clinical trials were considered.

#### 2.1.2 Horizontal bone augmentation with the staged approach

A primary search was performed on MEDLINE using the following combination of search terms: [“guided bone regeneration” OR “alveolar ridge augmentation” OR “bone augmentation”] AND [“autologous platelet concentrates” OR “platelet‐rich plasma” OR “plasma rich in growth factors” OR “platelet‐rich fibrin” OR “leukocyte‐ and platelet‐rich fibrin” OR “advanced platelet‐rich fibrin” OR “advanced platelet‐rich fibrin plus” OR “concentrated growth factor” OR “PRP” OR “PRGF” OR “L‐PRF” OR “A‐PRF” OR “A‐PRF+” OR “CGF”]. Due to the scarce number of publications about the topic, besides clinical studies, preclinical in‐vivo studies on large animals were also considered.

#### 2.1.3 Vertical bone augmentation with the staged approach

A primary search was performed on MEDLINE using the following combination of search terms: (((((((((((((((vertical bone regeneration AND platelet‐rich fibrin) OR (vertical bone regeneration AND platelet‐rich plasma)) OR (“vertical” [AIl Fields] AND “bone” [AIl Fields] AND “regeneration” [AIl Fields] AND (“plasma” [All Fields] AND “rich” [All Fields] AND “growth “[All Fields] AND “factors” [All Fields]))) OR (“vertical” [All Fields] AND “alveolar” [All Fields] AND “ridge” [All Fields] AND “augmentation” [All Fields] AND (“plasma” [All Fields] AND “rich” [AlI Fields] AND “growth” [All Fields] AND “factors” [AIl Fields]))) OR (vertical alveolar ridge augmentation AND platelet‐rich fibrin)) OR (vertical alveolar ridge augmentation AND platelet‐rich plasma)) OR (vertical bone augmentation AND platelet‐rich plasma)) OR (vertical bone augmentation AND platelet‐rich fibrin)) OR (“vertical” [All Fields] AND “bone” [All Fields] AND “augmentation” [AIl Fields] AND (“plasma” [All Fields] AND “rich” [All Fields] AND “growth” [AIl Fields] AND “factors “[All Fields]))) OR (“vertical” [All Fields] AND “ridge” [AIl Fields] AND “augmentation” [AIl Fields] AND (“plasma “[AlI Fields] AND “rich” [All Fields] AND “growth” [All Fields] AND “factors” [AIl Fields]))) OR (vertical ridge augmentation AND platelet‐rich fibrin)) OR (vertical ridge augmentation AND platelet‐rich plasma)) OR ((“Alveolar Ridge Augmentation” [Mesh]) AND “Platelet‐Rich Plasma” [Mesh])) OR ((“Alveolar Ridge Augmentation” [Mesh]) AND “Platelet‐Rich Fibrin” [Mesh])) OR ((“Bone Regeneration” [Mesh]) AND “Platelet‐Rich Plasma” [Mesh])) OR ((“Bone Regeneration” [Mesh]) AND “Platelet‐Rich Fibrin” [Mesh]). Due to the scarce number of publications about the topic, preclinical in‐vivo studies considering the mandible of large animals only as an experimental model and clinical studies were included.

The titles and abstracts (when available) of all identified studies were screened and imported into a database in the three clinical scenarios. Only papers written in English were considered. If articles appeared to meet the inclusion criteria or had insufficient data in their title and abstract, the full text was obtained to make the final decision. All studies that met the inclusion criteria underwent data extraction. Moreover, the references of the identified papers were screened for additional studies.

## 3 RESULTS

### 3.1 Horizontal/vertical bone augmentation with the simultaneous approach

A total of 1298 potentially eligible articles were identified through database searching. The title and abstract reading led to the exclusion of 1279 articles. After reviewing the remaining 19 full‐text articles and applying the eligibility criteria, 12 more publications were excluded. Finally, seven studies on horizontal or vertical GBR that used APCs were included for qualitative analysis.

### 3.2 Data analysis of the included studies (Table 1)

**TABLE 1:** Clinical studies exploring autologous platelet concentrates' benefits for simultaneous horizontal/vertical bone augmentation and implant placement.

Article	Study type; number and location of sites; type of GBR; defect classification^a^	Number (*n*), gender, and age of subjects	Centrifuge; rpm and minutes	GBR technique	Observations of horizontal bone gain (*other parameters*)
Sticky bone					
PRP/PRGF					
Iglesias‐Velázquez et al.^39^	RCT (pilot study); 9 sites at LJ; horizontal GBR; Class 2	*n* = 9 ♀ = 6/♂ = 3 age: 49–72 y	System V, BTI L: 580 g, 8 min	T (6): PRGF + Bio‐Oss + autologous bone + PPF C (3): Bio‐Oss + absorbable membrane (CopiOs) + pins	5 months after GBR, similar total horizontal bone width: T: 9.4 ± 1.8 mm; C: 9.3 ± 0.4 mm postoperative pain: **T: 83% mild, 17% moderate; C: 100% moderate**
Liquid fibrinogen/i‐PRF					
Işik et al.^40^	RCT; 40 sites at LJ (posterior); horizontal and vertical GBR; Class 2	*n* = 40 ♀ = 22/♂ = 18 age: (T): 50 ± 7.7 y (C): 48 ± 7.7 y	EBA 200, L: 700 rpm, 3 min	T (20): liquid PRF+ Bio‐Oss + Bio‐Gide membrane C (20): Bio‐Oss + Bio‐Gide membrane	6 months after GBR, horizontal bone gain: coronally, T: **1.6 ± 0.2**, C: 1.3 ± 0.1 mm medially, T: **2.6 ± 0.3**, C = 2.5 ± 0.2 mm apically, T: **3.1 ± 0.4**, C: 3.0 ± 0.2 mm
Wang et al.^41^	Prospective cohort study (conventional vs. digital workflow, 1 GBR procedure); 14 sites at LJ and UJ (anterior); horizontal GBR; Class 2	*n* = 14 ♀ = 7/♂ = 7 mean age: 42 ± 13 y	Trausim AiPRF‐08 L: 700 rpm, 3 min	Mix: i‐PRF + Bio‐Oss + Bio‐Gide + fixation pins	6 months after GBR, horizontal bone gain (*per mm apically from shoulder*): *Conventional group 0*: 0.9 ± 0.5 mm, *1*: 1.3 ± 0.7 mm, *2*: 1.7 ± 0.5 mm *3*: 2.2 ± 0.8 mm, *4*: 2.3 ± 1.0 mm, *5*: 2.2 ± 1.1 mm *Digital group: 0*: 1.9 ± 0.6 mm, *1*: 2.2 ± 0.6 mm, *2*: 2.4 ± 0.9 mm *3*: 2.9 ± 0.9 mm, *4*: 2.9 ± 1.1 mm, *5*: 3.1 ± 1.1 mm
L‐PRF bone‐block variants					
Dai‐Y et al.^42^	Retrospective cohort study; 29 sites at UJ/LJ; horizontal GBR; Class 2	*n* = 29 ♀ = 16/♂ = 13 age: 20–58 y	Medifuge # settings.	T (14): CGF ^cl^ + mineralized collagen (1:1 ratio) + membrane C (15): mineralized collagen + membrane	6 months after GBR, horizontal bone gain: coronally, T: **2.4 ± 0.8 mm**, C: 2.1 ± 0.5 mm medially, T: **1.3 ± 0.8 mm**, C: 1.2 ± 0.4 mm apically, T: **1.29 ± 0.36**, C: 1.25 ± 0.35 mm (T): **superior PROMs**
Al‐Aroomi et al^43^	Retrospective cohort study (1 arm); 28 sites at UJ (anterior and posterior); horizontal GBR; Class 1 and 2	*n* = 28 ♀ = 6/♂ = 22 mean age: 43 ± 8 y	Not mentioned	Mix: A‐PRF ^cl^ + Bio‐Oss + Bio‐Gide	6–7 months after GBR, horizontal bone gain at the anterior maxilla: 2.5 ± 1.1 (implant neck), 4.0 ± 1.4 (coronal third), 4.2 ± 1.5 (middle third), and 4.1 ± 1.5 (apex implant) 18–24 months after GBR, horizontal bone gain at the anterior maxilla: 2.4 ± 1.1 (implant neck), 3.7 ± 1.2 (coronal third), 4.0 ± 1.4 (middle third), 4.0 ± 1.6 (apex implant)
Caramês et al.^44^	Prospective case series; 72 sites at UJ (anterior); horizontal GBR; Class 2 and 3	*n* = 18 ♀ = 13/♂ = 5 age: 36–76 y	IntraSpin Liquid fibrinogen: 2700 rpm, 3 min L‐PRF membranes: 2700 rpm, 12 min	Mix: liquid fibrinogen + chopped L‐PRF ^m^ + Bio‐Oss + autologous bone and L‐PRF ^m^ as a barrier membrane	12 months after GBR, horizontal bone gain: implant neck: 3.0 mm (2.5–3.5) mid‐implant: 3.5 mm (2.9–4.1)
De Angelis et al.^45^	Retrospective cohort study; 20 sites at UJ/LJ; horizontal and vertical GBR; Class 2	*n* = 20 ♀ = 11/♂ = 9 mean age: 60 ± 10 y	EBA 200, M: 1500 rpm, 14 min L: 700 rpm, 3 min	T: Mix: i‐PRF + A‐PRF + xenograft (50:50) + resorbable membrane fixed with pins C: autogenous bone + xenograft + resorbable membrane fixed with pins	9 months after GBR, the predictable procedure for closing favorable three‐dimensional peri‐implant defects. Mean peri‐implant vertical defect heights at baseline were T: 4.0 ± 1.5 mm and C:3.6 ± 0.9 mm No SS differences in the mean residual defect (T: 0.1 ± 0.5 mm and C: 0.2 ± 0.6 mm). SS better wound healing in group B (1st week)

*Note*: Data in bold reached statistical significance between test and control sites if present. Abbreviations: A‐PRF, advanced PRF; C, control; CGF, concentrated growth factors; ^cl^, clot; g, g‐force; GBR, guided bone regeneration; i‐PRF, injectable PRF; L, liquid form; LJ, lower jaw; L‐PRF, leucocyte‐ and platelet‐rich fibrin; ^m^, membrane; M, solid form; min, minutes; mix, mixture; PPF, Periosteal pocket flap technique; PRGF, plasma rich in growth factors; PROMs, patient‐reported outcome measures; PRP, platelet‐rich plasma; RCT, randomized clinical trial; rpm, revolutions per minute; SS, statistically significant; T, test; UJ, upper jaw; y, year.

^a^ Defect classification based on Benic and Hämmerle.^14^

#### 3.2.1 Sticky bone

In a randomized clinical trial (pilot study, nine patients) by Iglesias‐Velázquez et al.,^39^ the clinical and radiographic outcome of a periosteal pocket flap technique using xenogeneic plus autologous bone and liquid PRGF (six patients) was compared to a conventional GBR procedure (three patients). It revealed no significant differences in horizontal bone gain, surface area, and volume between the two groups. However, the PRGF patients obtained better results regarding postoperative pain.

Işik et al.^40^ in another randomized clinical trial, assessed bone augmentation success after simultaneous GBR and implant placement in the posterior mandible using bovine‐derived xenograft alone versus combined with a liquid PRF. That study included 20 patients with 50 implants in the test group (xenograft + liquid PRF) and 20 with 48 implants in the control group (xenograft alone). The authors found small but statistically significant differences in horizontal bone gain favoring the addition of liquid PRF. No significant differences were found between the test and control groups regarding implant survival and peri‐implant marginal bone loss.^40^

A prospective cohort study by Wang et al.^41^ evaluated the impact of i‐PRF (injectable PRF) by producing a shapable PRF block to regenerate anterior Class 2 defects using a digital workflow versus a conventional workflow, with the same GBR technique in both groups. The buccal bone gain was assessed at every millimeter apically from the implant shoulder in both groups. After 6 months, the bone gain with the conventional workflow was *0*: 0.9 ± 0.5 mm, *1*: 1.3 ± 0.7 mm, *2*: 1.7 ± 0.5 mm, *3*: 2.2 ± 0.8 mm, *4*: 2.3 ± 1.0 mm, and *5*: 2.2 ± 1.1 mm, and with the digital workflow was *0*: 1.9 ± 0.6 mm, *1*: 2.2 ± 0.6 mm, *2*: 2.4 ± 0.9 mm, *3*: 2.9 ± 0.9 mm, *4*: 2.9 ± 1.1 mm, *5*: 3.1 ± 1.1 mm, apically from the shoulder, respectively.^41^

#### 3.2.2 L‐PRF bone block

In a retrospective cohort study, 29 patients received one of two regeneration approaches in Class 1 and 2 defects: GBR with a particulate alloplastic material alone or mixed with CGF (concentrated growth factor) clots in a 1:1 ratio. The results indicated that the mean graft thickness decreased significantly in the first 3 months with both approaches, remaining stable between 3 and 6 months. Importantly, the new buccal bone was significantly thicker in the CGF group at 3 and 6 months.^42^

In another retrospective cohort study by Al‐Aroomi et al.^43^ with only one arm for GBR, the impact of using demineralized bovine bone mixed with advanced PRF (A‐PRF) was tested in immediate implant placement. The horizontal bone gain was measured at four locations: the implant neck, the coronal third, the middle third, and the implant apex. At 6 months, after immediate implant placement, the bone gain in the anterior maxilla was 2.5 ± 1.1 at the implant shoulder level and 4.0 ± 1.4, 4.2 ± 1.5, and 4.1 ± 1.5 in subsequent apical measurements. In the same study, another group of patients was treated with delayed implant placement (i.e., after alveolar ridge preservation). Throughout the follow‐up periods, the authors observed slightly more facial bone gain in the immediate implant placement group compared to the delayed placement group.

**FIGURE 3:**
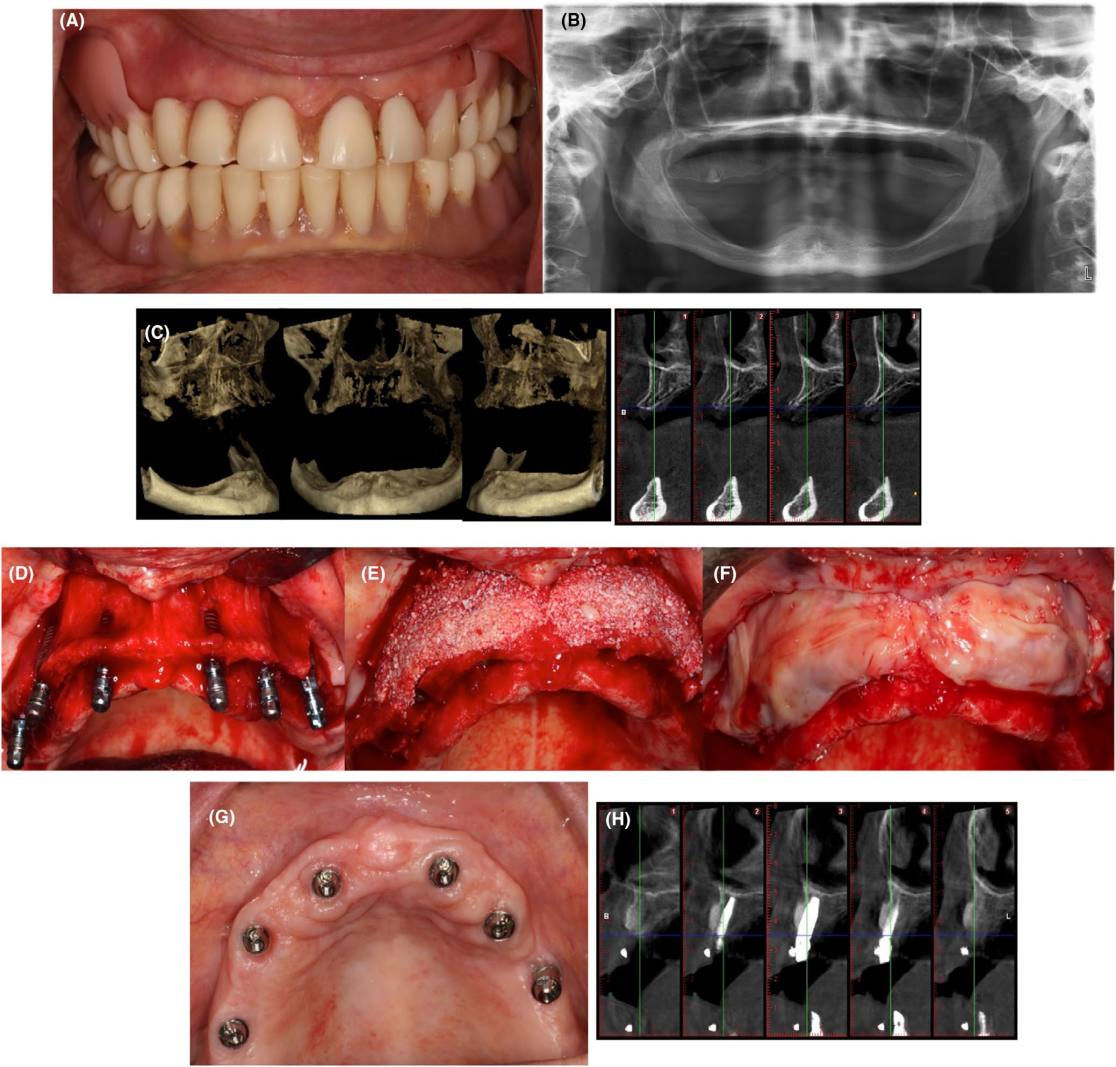
Clinical case of simultaneous horizontal bone augmentation and implant placement. (A) 55‐year‐old female patient had a fully edentulous upper arch with poor bone quantity and quality (Class 3). (B) Preoperatory panoramic image. (C) Preoperatory cone‐beam computed tomography (CBCT). (D) Surgical approach for implant placement. (E and F) Horizontal bone augmentation with leukocyte‐ and platelet‐rich plasma (L‐PRF) bone block (xenograft, chopped pieces of two L‐PRF membranes, and liquid fibrinogen) covered with L‐PRF membranes. (G) 1‐year follow‐up. (H) 1‐year follow‐up CBCT.

The potential benefit of L‐PRF associated with a xenograft in horizontal GBR was also studied by Caramês et al.^44^ Their prospective case series study (Figure 3) included 18 edentulous patients with Class 2 and 3 horizontal ridge deficiencies in the anterior maxilla who were candidates for full‐arch rehabilitation. Horizontal linear measurements from consecutive cone‐beam computed tomography (CBCT) scans obtained at presurgery, postsurgery, and the 12‐month follow‐up were assessed. The mean horizontal bone width was 4.5 (4.1–4.8) mm presurgery, 9.3 (8.8–9.6) mm immediately after GBR, and 7.7 (7.3–8.1) mm at the 12‐month follow‐up.

A retrospective cohort study by De Angelis et al.^45^ evaluated the clinical and radiographic outcomes of simultaneous horizontal and vertical GBR using either a bone xenograft mixed with autogenous bone + collagen membrane (Group A) or a bone xenograft mixed with A‐PRF + collagen membrane (Group B). The mean peri‐implant vertical defect heights at baseline in groups A and B were 3.6 ± 0.9 mm and 4.0 ± 1.5 mm, respectively (*p* = 0.382). There were no statistically significant differences in the mean residual defect heights: 0.2 ± 0.6 mm for group A and 0.1 ± 0.5 mm for group B. The mean residual defect widths of the two groups also showed no statistically significant difference. However, after the first week, statistically significant differences in postoperative wound healing were observed, with a trend toward greater healing in group B.

### 3.3 Horizontal bone augmentation with the staged approach

A total of 1893 potentially eligible articles were identified through database searching. The title and abstract reading led to the exclusion of 1855 articles. Of the remaining 38 papers, 26 were excluded after full‐text screening and eligibility criteria application. Thus, 12 studies remained for data extraction: eight animal studies and four clinical trials.

#### 3.3.1 Animal studies on a mixture of a particulate bone substitute and an APC

Kurikchy et al.^46^ compared the bone healing between three intrabony defects in the femur bone of 16 rabbits: one was left unfilled for unassisted healing, one was filled with a xenogenic graft, and the other was filled with the same xenograft mixed with autologous PRP. They observed that adding PRP enhanced new bone formation and neovascularization significantly.

Chen et al.^47^ assessed the bone healing of one circular mandibular bi‐cortical bony defect (54 rabbits), comparing unassisted healing with the application of porous bone mineral mixed or not with autologous PRP covered with a collagen membrane. The addition of PRP resulted in more new bone formation, more bone filled, and increased osteoblast activity.

Oliveira et al.^48^ explored the impact of adding chopped L‐PRF membrane pieces to a deproteinized bovine bone matrix (DBBM) during the regeneration of critical‐size, well‐contained, bony defects in the calvaria of rats. They reported significantly more bone regeneration with the L‐PRF + DBBM mixture (54% after 30 days and 64% after 60 days) than with DBBM alone (26% and 57%, respectively).

Engler‐Pinto et al.^49^ repeated the study on rats with osteoporosis induced by ovariectomy to mimic a more compromised condition. Again, the L‐PRF + DBBM mixture resulted in more new bone formation (28% vs. 21%). They considered that the improved outcome when applying L‐PRF to DBBM resulted from: (a) the presence of hematopoietic stem cells, platelets, leukocytes, and fibroblasts in L‐PRF; (b) the release of large amounts of cytokines and growth factors by L‐PRF, including VEGF, which is the most important growth factor for angiogenesis regulation; (c) L‐PRF's typical 3D fibrin matrix with an elastic architecture and specific composition that favors cell migration and molecule retention; and (d) the fact that L‐PRF improves the osteoconductive potential of bovine bone grafts.

Yoon et al.^50^ used a similar protocol for critical‐size cranial defects in rabbits. Follow‐up biopsies at 1, 2, and 4 weeks revealed a significantly higher immunostaining intensity for VEGF with the L‐PRF + DBBM mixture.

Şimşek et al.^51^ created bone defects (9‐mm diameter, 4‐mm depth) around 3‐mm diameter implants in rabbit tibias and grafted them with one of the following three mixtures: demineralized freeze‐dried bone allograft (DFDBA) plus saline solution, DFDBA plus rifamycin, and DFDBA plus L‐PRF. The histology after 4 weeks of healing showed the following bone‐to‐implant contacts: 51% in the DFDBA+saline solution group, 60% in the DFDBA+rifamycin group, and 73% in the DFDBA+L‐PRF group. The percentage of new bone formation at the defect area was 38%, 49%, and 63%, respectively.

You et al.^52^ analyzed the healing of 8‐mm diameter femoral defects in dogs, comparing unfilled defects, defects filled with deproteinized bovine bone mineral (DBBM) alone, or DBBM mixed with L‐PRF. Both radiographical and histological evaluations showed more new bone formation at 2 weeks (but no longer after 4 weeks) in defects filled with the L‐PRF + DBBM mixture compared to the other approaches.

Rezuc et al.^53^ prepared four bone defects in the tibiae of 12 rabbits and filled each with DBBM, L‐PRF, or a combination of DBBM plus L‐PRF or left them to heal unassisted as a control site. All defects were covered with a collagen membrane. The use of L‐PRF alone or combined with DBBM did not show any additional histological improvement regarding the amount of vital bone formation compared to DBBM alone.

Park et al.^54^ compared the healing of surgically created two‐wall defects in beagle dogs between three experimental groups: defect filled with sticky bone (a mixture of deproteinized porcine bone mineral (DPBM) and i‐PRF), defect filled with sticky bone covered by an A‐PRF membrane, and defect filled with DPBM covered by a collagen membrane. Neither Micro‐CT nor histological analyses indicated significant differences between the three groups. Consistently, graft consolidation, as indicated by new bone formation at the defect site, did not differ significantly between groups.

The outcome of the animal studies is conflicting, but most of them indicate that combining a bone substitute (DBBM or DFDBA) with L‐PRF facilitates bone regeneration.

#### 3.3.2 Clinical studies on the use of sticky bone or an L‐PRF bone block (Table 2)

**TABLE 2:** Randomized clinical trials and prospective cohort studies exploring autologous platelet concentrates' benefits for staged horizontal bone augmentation.

Article	Study type; number and location of sites; type of GBR; defect classification^a^	Number (*n*), gender, age, and smoking habits of subjects	Centrifuge; rpm and duration	GBR technique	Observations of horizontal bone gain (*other parameters*)
Sticky bone					
PRP/PRGF					
Eskan et al.^55^	RCT, parallel; 28 sites at UJ/LJ; horizontal GBR; Seibert Class 1 or 3	*n* = 28 ♀ = 14/♂ = 14 age: 19–75 y non‐smokers	Haverst Technologis NR	Mix: cancellous allograft + bone screw + polylactide ^membr^ C (14): no PRP T (14): + PRP plasma	Comparison before vs. 4 months after GBR: C: gain^0/5 mm from crest^ = 2.0 ± 1.2/2.2 ± 1.3 mm, T: gain^0/5 mm from crest^ = **2.9 ± 1.0**/1.9 ± 1.5 mm, **T: > % vital bone (51 ± 15 vs. 36 ± 14%)**
Liquid fibrinogen/i‐PRF					
Amaral Valladao et al.^56^	Retrospective case series; 29 sites at UJ/LJ; (1 arm) horizontal GBR; Cawood and Howell class IV	*n* = 10 ♀ = 6/♂ = 4 mean age: 59.4 y non‐smokers	IntraSpin L: 700 rpm, 3 min	Mix: Bio‐Oss + particulate autogenous bone + i‐PRF + collagen^membr+tags^ + L‐PRF ^m to cover^	7.5 ± 1 month after GBR (at re‐entry): mean gain^5/7/11 mm from crest^=5.9 ± 2.4 mm
Tony et al.^58^	RCT, parallel; 20 sites at UJ/LJ; horizontal GBR; Kennedy class 3	*n* = 20 ♀ = 10/♂ = 10 mean age: 36.1 y non‐smokers	Dentifuge LC‐100 L: 700 rpm, 3 min	Mix: Bio‐Oss + i‐PRF C (10): + collagen^membr+tags^ T (10): + no membrane	Comparison before vs. 6–8 months after GBR: C: gain^0/3/6 mm from crest^ = 1.4/1.4/2.0 mm T: gain^0/3/6 mm from crest^ = **2.7/2.8/2.6** mm
Aboelela et al.^57^	RCT, parallel; 28 sites at UJ/LJ; horizontal GBR; Len class IV	*n* = 28 ♀ = 12/♂ = 16 mean age: 40.2 y NR	NR L: 2500 rpm, 3 min M: # settings	Mix: Bio‐Oss + particulate autogenous bone: C (14): + collagen^membr+tags^ T (14): + autologous fibrin glue + CGF ^membr to cover^	Comparison before vs. 6 months after GBR: C: mean gain^2/5/10 mm from crest^ = **2.2 mm** T: mean gain^2/5/10 mm from crest^ = 1.8 mm
L‐PRF bone block					
Cortellini et al.^59^	Prospective case series; 10 sites at UJ; horizontal GBR; Benic & Hämmerle Class 1–3	*n* = 10 ♀ = 6/♂ = 4 age: 23–72 y, NR	IntraSpin L: 2700 rpm, 3 min M: 2700 rpm, 12 min	Mix: Bio‐Oss + liquid fibrinogen + L‐PRF ^chopped membr^. + collagen^membr+tags^ + L‐PRF^m final cover^	6–8 months after GBR (at re‐entry): gain^2/6 mm from crest^ = 4.6 ± 2.3/5.3 ± 1.2 mm resorption^2/6 mm from crest^ 16/11%
Cortellini et al.^60^	Prospective case series; 36 sites at UJ; horizontal GBR; Benic & Hämmerle Class 1–3	*n* = 29 ♀ = 17/♂ = 12 mean age: 51 ± 17 y NR	IntraSpin L: 2700 rpm, 3 min M: 2700 rpm, 12 min	Mix: Bio‐Oss + liquid fibrinogen + L‐PRF^chopped membr^. + collagen ^membr+tags^ + L‐PRF ^m final cover^	6–8 months after GBR (at re‐entry): gain 4^mm from crest^ = 4.6 ± 1.3 mm

*Note*: Data in bold reached statistical significance between test and control sites if present. Abbreviations: C, control; CGF, concentrated growth factors; GBR, guided bone regeneration; i‐PRF, injectable PRF; L, liquid form; LJ, lower jaw; L‐PRF, leucocyte‐ and platelet‐rich fibrin; M, solid form; ^membr^, membrane; min, minutes; mix, mixture; NR, Not reported; PRGF, plasma rich in growth factors; PRP, platelet‐rich plasma; RCT, randomized clinical trial; rpm, revolutions per minute; T, test; UJ, upper jaw; y, year.

^a^ Defect classification based on Benic and Hämmerle.^14^

Only five clinical trials (three randomized clinical trials (RCTs) and two prospective cohort studies) focused on a horizontal bone augmentation combined with staged implant placement applying an APC.

##### Sticky bone

Four clinical trials analyzed the impact of sticky bone on horizontal bone augmentation. An RCT by Eskan et al.^55^ explored the added value of applying PRP (liquid form, mixed with substitute) during horizontal bone augmentation using a cancellous allograft, bone screws, and a polylactide membrane as cover. The addition of PRP resulted in significantly more horizontal bone gain (2.9 vs. 2.0 mm) at the 4‐month re‐entry. In most patients, a bone core was taken for histological analyses. PRP sites showed significantly higher percentages of vital bone (51 vs. 36%).

Amaral Valladao et al.,^56^ in their retrospective case series study, obtained a mean horizontal alveolar bone width gain of 5.9 ± 2.4 mm when using the combination of particulate autologous bone and xenogenous graft (1:1), i‐PRF to agglutinate the graft, and an absorbable collagen membrane (+ tags), covered with L‐PRF membranes.

Aboelela et al.^57^ compared a 1:1 mixture of particulate autogenous bone and anorganic bovine bone mineral covered with a collagen membrane (control group) with a similar mixture that also included an autologous fibrin glue to make a sticky bone and was covered with a CGF membrane (test group). The latter resulted in significantly less bone gain (1.8 vs. 2.2 mm), probably due to fast resorption of the CGF membrane.

Tony et al.^58^ examined the need for a collagen membrane (and tags) on top of the sticky bone and observed significantly more bone gain (at the coronal part of the graft (2.9 vs. 2.0 mm) up to 5 mm apically), at sites without a collagen membrane compared to sticky bone covered with a barrier membrane.

##### L‐PRF bone block

**FIGURE 4:**
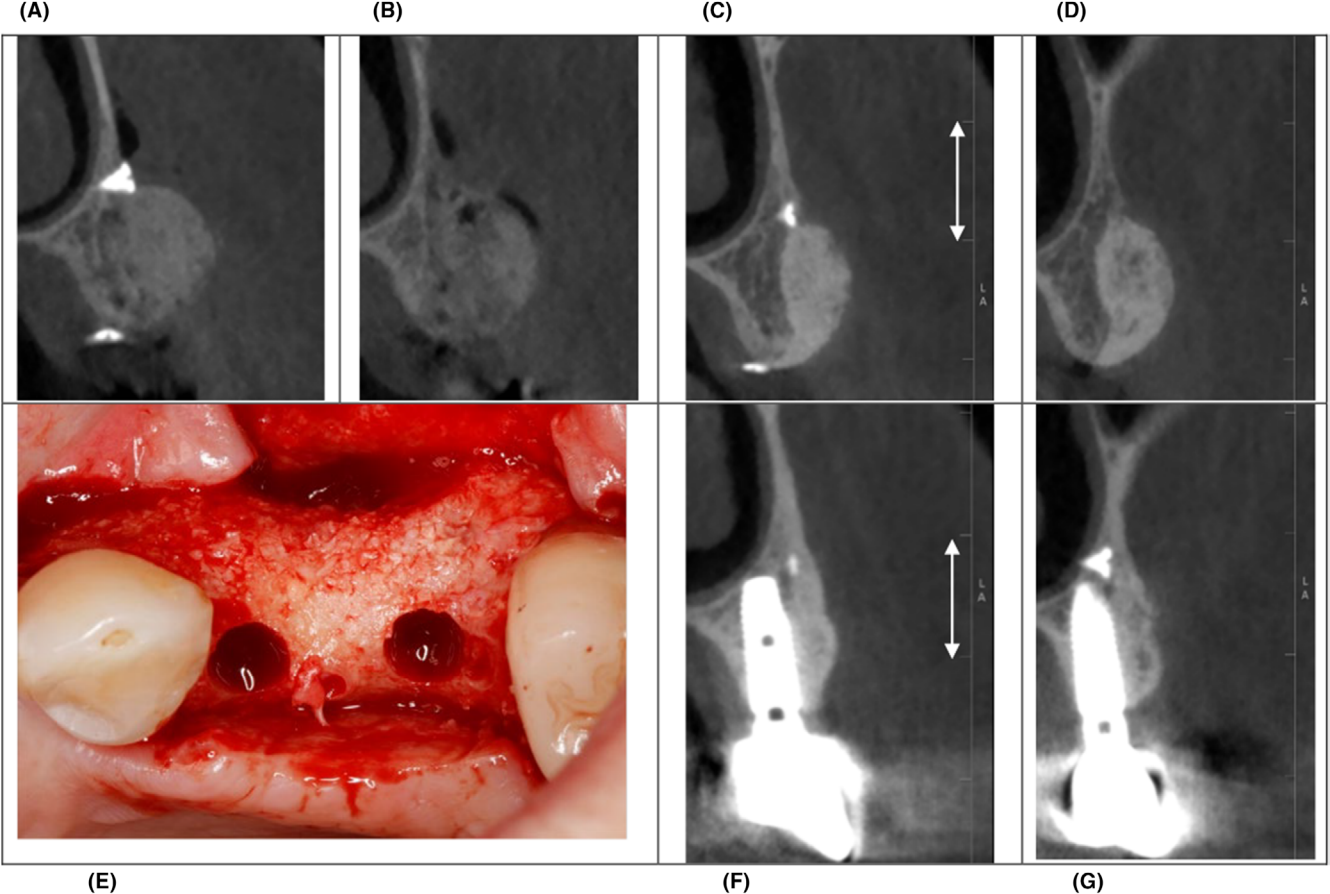
Clinical case of a staged horizontal bone augmentation via a leucocyte‐ and platelet‐rich fibrin (L‐PRF) bone block. (A and B) The patient and the referring dentist insisted on installing two implants (instead of one implant and one cantilever) to replace missing 24 and 25 teeth. Cross‐sectional cone‐beam‐computed tomography images obtained immediately after guided bone regeneration (GBR) indicated an initial alveolar ridge width of ±4 mm and a horizontal bone augmentation of 5 (24) and 7 (25) mm. (C and D) Healing after 8 months confirmed a nice bone gain with a significant increase in radiopacity and a filling of open areas (probably representing larger pieces of chapped L‐PRF). (E): Re‐entry 8 months after horizontal GBR showed a dense bone, without loose Bio‐Oss particles, and well‐vascularized osteotomies. (F and G) Cross‐sectional CBCT images in the middle of each implant, taken after 1.5 years of implant loading. Over time, early and late graft resorption is observed, a standard observation after horizontal GBR (for details, see Quirynen et al.^11^).

A first clinical trial on L‐PRF bone blocks (prepared as a mixture of a bone substitute (Bio‐Oss), liquid fibrinogen, and pieces of chopped L‐PRF membranes (50/50% substitute/chopped membrane), covered with a collagen membrane overlayed with L‐PRF membranes) included 15 sites with lateral alveolar deficiencies in need of augmentation prior to implant placement.^59^ Superimposition of preoperative and posthealing CBCT scans (after 6–8 months) showed an average linear lateral bone gain of 4.6 mm (±2.3), 5.3 mm (±1.2), and 4.4 mm (±2.3), measured at 2, 6 and 10 mm from the alveolar crest, respectively. The volumetric gain was 1.05 cm^3^ (±0.7) on average. All implants inserted in the regenerated bone were well integrated and are still in function after 2 years of loading. In the meantime,^60^ 29 patients were treated at the Department of Periodontology in Leuven, with similar results (Figure 4). The horizontal alveolar bone width gain, 4 mm away from the bony crest, was 4.6 ± 1.3 mm. A significant increase in radiopacity was systematically observed when comparing the follow‐up CBCT with the CBCT obtained immediately after applying the L‐PRF bone block. It is plausible to assume that a major part of the L‐PRF is replaced by osseous tissue, but histological analysis of biopsies will be needed to confirm this hypothesis.

Fang et al.^61^ conducted an RCT to examine the beneficial impact of adding CGF clots to a Bio‐Oss bone powder (test group) compared to Bio‐Oss bone powder alone to fill different types of bony defects (extraction of impacted teeth, removal of jaw cyst or tumor, jawbone fracture). CBCT analyses showed significantly higher bone mineral densities in the test group 1 week, 12 weeks, and 6 months after surgery. They also observed bone formation in the jaw defect area separated from the surrounding jaw bones in the test sites and not in the control sites. This finding illustrates the osteo‐inductive capacities when second‐generation APCs are added to a bone substitute.

### 3.4 Vertical bone augmentation with the staged approach

A total of 607 potentially eligible articles were identified through database searching. The title and abstract reading led to the exclusion of 586 articles. After reading the remaining 21 full‐text articles and applying the eligibility criteria, 17 other publications were excluded. Four studies on vertical bone augmentation with the staged approach and APCs were included for qualitative analysis: two preclinical in‐vivo studies performing vertical ridge augmentation in the mandible of dogs and two clinical studies (one RCT and one retrospective cohort (one arm) study).

#### 3.4.1 Preclinical in‐vivo studies

##### Sticky bone

Park et al.,^54^ in a preclinical in‐vivo study (beagle dog), evaluated the impact of sticky bone prepared using L‐PRF and DPBM in combination with L‐PRF membranes on the regeneration of two‐wall damaged extraction sockets (the buccal and lingual walls of the sockets were eliminated, similar to a vertical regeneration). Three damaged extraction sockets were randomly allocated to the following groups: group 1, where the socket was grafted with DPBM and covered with a collagen membrane (positive control); group 2, where the socket was filled with DPBM and the liquid L‐PRF mixture; group 3, where the socket was filled with sticky bone and then covered with an L‐PRF membrane. There were no significant differences between the groups in terms of augmented and regenerated ridge area. However, in the gene expression analysis performed after 1 week of healing, greater quantities of favorable signaling molecules (BMP‐2, IL‐6, VEGF, osteocalcin, and calcitonin receptor) were found in the L‐PRF groups compared to the GBR group.

##### L‐PRF membranes (Figure 5)

**FIGURE 5:**
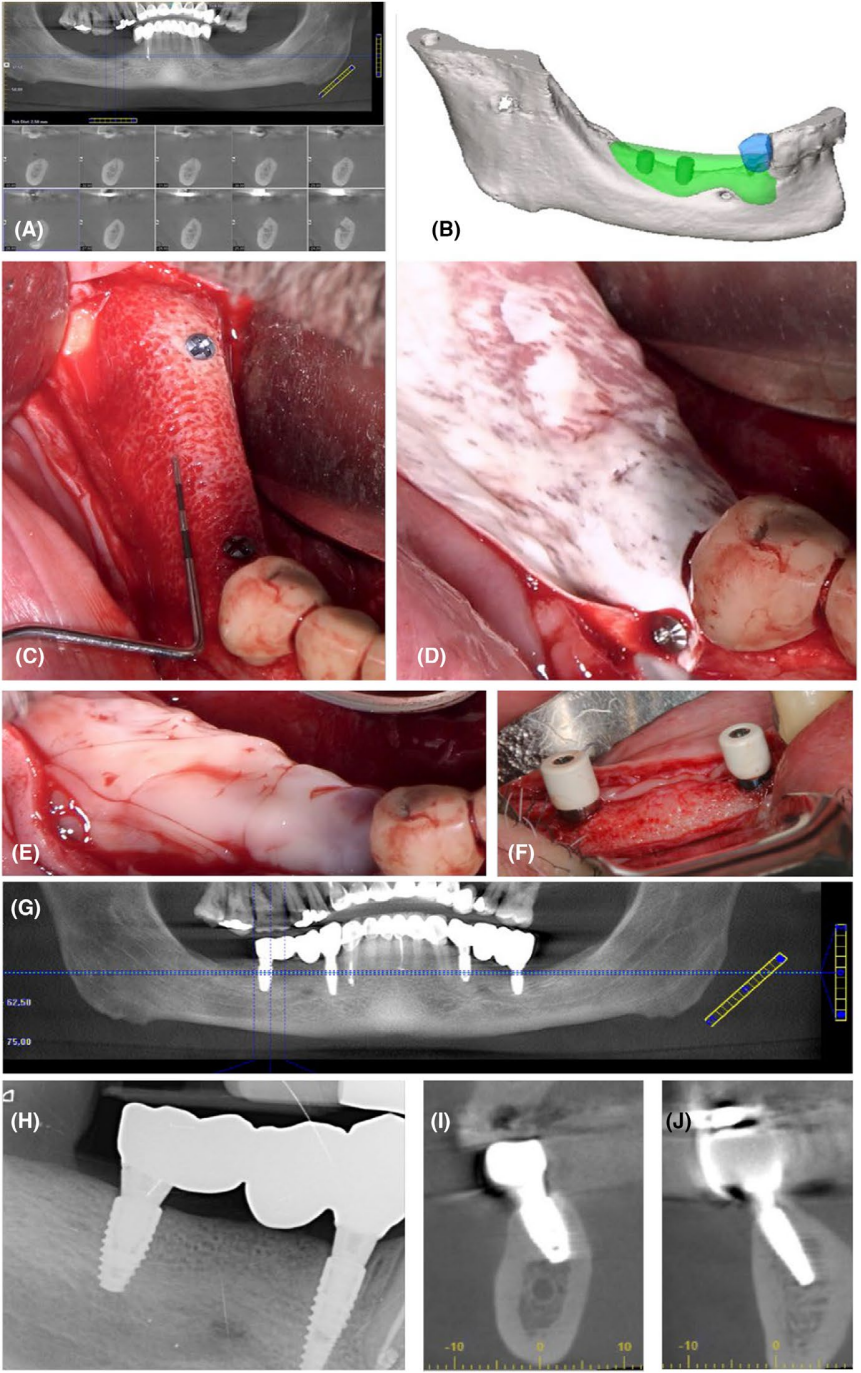
Clinical case of a staged vertical bone augmentation via a CAD/CAM allograft scaffold plus collagen membrane covered with a leucocyte‐ and platelet‐rich fibrin (L‐PRF) membrane. (A) Partially edentulous patient with little bone quantity in the posterior lower jaw, particularly on the right side. (B) CAD/CAM design of the allograft scaffold for the right side. (C and D) Allograft scaffold fixed with two screws on top of the crestal bone and covered with a collagen membrane. (E) L‐PRF membranes covering the collagen membrane. (F) After 6 months of healing, two implants were installed (44, 46). (G) 1‐year follow‐up. (H–J) 2‐year follow‐up.

Eldabe et al.^62^ in a preclinical in‐vivo study (mongrel dog), analyzed the influence of collagen membrane perforations and L‐PRF for vertical ridge augmentation. This study was performed in standardized mandibular saddle defects (8–10 mm mesiodistal/6–8 mm apicocoronal/12–14 mm buccolingual). Defects were randomly allocated into four groups: 1, xenograft block + collagen membrane; 2, xenograft block + four L‐PRF membranes + collagen membrane; 3, xenograft block + perforated collagen membrane (PCM); and 4, xenograft block + four L‐PRF membranes + PCM. There were no statistically significant differences between groups regarding vertical bone gain and vertical bone resorption. However, the highest vertical bone gain and the lowest resorption were obtained in group 4 (block + L‐PRF + PCM). Group 4 also showed a significant difference compared to group 1 regarding the total bone percentage and bone maturation. Therefore, the use of xenogenic block grafts combined with double‐layered perforated collagen and L‐PRF membrane in vertical ridge augmentation appeared to improve the inductive power of this challenging defect type.

#### 3.4.2 Data analysis of the included clinical studies (Table 3)

**TABLE 3:** Clinical studies exploring the benefits of APCs for staged vertical bone augmentation.

Article	Study type; number and location of sites; type of GBR; defect class^a^	Number (*n*), gender, and age of subjects	Centrifuge; rpm and duration	GBR technique	Observations
PRGF					
Torres et al.^63^ (*vertical GBR only*)	RCT 43 sites at UJ/LJ; 3D augmentation (vertical/horizontal)	*n* = 30 ♀ = 17/♂ = 13 age 48–76 y	Bti PRGF System II	T (22): ABB + Ti‐mesh + PRGF plug C (21): ABB + Ti‐mesh	6 months after GBR, vertical bone gain: **T: 3.5 ± 0.7 mm, C: 3.1 ± 0.8 mm** Ti‐mesh exposure: **T: 0/22 C: 6/21**
Sticky bone					
Amaral Valladao et al.^56^ (*vertical GBR only*)	Retrospective case series; 23 sites at UJ/LJ; Cawood and Howell Class 5	*n* = 8 ♀ = 5/♂ = 3 mean age: 55.4 y	IntraSpin i‐PRF: 2700 rpm, 3 min L‐PRF^m^: 2700 rpm, 12 min	Mix: Bio‐Oss + particulate autogenous bone + i‐PRF + d‐PTFE^membr+tags^ + L‐PRF^*m*^	7.5 ± 1 month after GBR, vertical bone gain: 5.6 ± 2.6 mm no complications

*Note*: Data in bold reached statistical significance between test and control sites if present. Abbreviations: ABB, anorganic bovine bone; C, control; GBR, guided bone regeneration; i‐PRF, injectable PRF; LJ, lower jaw; L‐PRF, leucocyte‐ and platelet‐rich fibrin; ^m^, membrane; min, minutes; mix, mixture; PRGF, plasma rich in growth factors; RCT, randomized clinical trial; rpm, revolutions per minute; T, test; Ti, titanium; UJ, upper jaw; y, year.

^a^ Defect classification based on Benic and Hämmerle.^14^

##### Sticky bone

Torres et al.^63^ in a randomized clinical trial, addressed the efficacy of PRP in preventing Ti‐mesh exposure in cases of alveolar ridge augmentation (3D augmentation, with anorganic bovine bone covered with a Ti‐mesh with/without PRP on top). They treated 30 patients (15 with PRP and 15 without PRP). The Ti‐mesh was exposed in six cases (28.5%) of the non‐PRP group and none of the PRP group. The mean alveolar bone height gain was 3.1 ± 0.8 mm and 3.5 ± 0.7 mm for the non‐PRP and PRP groups, respectively, being statistically significant. Therefore, applying PRP over a Ti‐mesh may prevent complications such as mesh exposure and graft failure and improve the results in terms of bone regeneration.

Valladao et al. (2020), in a pre–post retrospective clinical study (one arm), evaluated the vertical bone gain using GBR (with sticky bone and non‐resorbable membrane covered with an L‐PRF membrane) in eight patients (23 sites, maxilla, and mandible) in Cawood and Howell class V defects. The follow‐up ranged from 1 to 7 years. After a healing period of 7.5 ± 1 months, they obtained a mean vertical bone gain of 5.6 ± 2.6 mm. All cases evolved without complications (e.g., infection, membrane exposure, and wound dehiscence), and implants could be installed in all cases. No differences were found between the sites (maxilla/mandible; anterior/posterior).

In summary, the three reviews gathered a total of 14 clinical studies assessing the additional benefits of APCs in alveolar bone augmentation. Among these, eight were controlled studies (six RCTs and two retrospective cohort studies), while the remaining six lacked a control group (four case series and two cohort studies in one arm). In four out of the six RCTs, the comparison was between the “standard technique” (biomaterial + collagen membrane) and the new approach (biomaterial + APC + collagen membrane), and in three the result was statistically significant in favor of the APC groups (the additional bone gain was between 0.3–0.9 mm), while the fourth RCT, a pilot study with only nine patients (six in the test group and three in the control group), showed no difference between groups. Therefore, considering these four RCTs, APCs seem to provide a clear benefit in alveolar bone augmentation. In the remaining two RCTs, the comparison was between using or not using a collagen membrane to cover the APC bone block, yielding controversial results. In the two retrospective cohort studies, which mirrored the comparison in the initial four RCTs, the results were statistically significant in one study and did not demonstrate differences in the other. However, the latter exhibited a positive trend favoring the APC group. All case series presented results consistent with the existing literature regarding bone augmentation in alveolar bone. Therefore, the following benefits of using the APCs in comparison with other biomaterials for bone augmentation were observed: additional bone gain, better manageability and graft stability, fewer bone substitute particles, better patient‐related outcomes (less pain, better wound healing, and less inflammation), and lower costs.

## 4 DISCUSSION

The results from the three present narrative reviews demonstrate that there are very few studies regarding the benefit of APCs in bone augmentation, both in the simultaneous and staged approaches. This procedure is in a very early stage of evidence. However, the current research is very promising, suggesting a positive benefit of this biomaterial in this clinical scenario, not only for better soft‐tissue healing in bone augmentation procedures but also for bone healing.

The review of the horizontal/vertical simultaneous approach analyzed seven clinical studies. Four had a control group (one RCT, one RCT‐pilot study, and two retrospective cohort studies), of which two^40^, ^42^ found statistically significant better bone augmentation in the APC groups, while the other two found no statistical differences but better tendencies in the APC groups.^39^, ^45^ Moreover, the APC groups obtained better results in terms of pain control, inflammation, or swelling.^39^ The results of the remaining studies (one case series and two cohort studies with only one arm of GBR) were very similar to what is currently published in the literature about horizontal bone gain.^7^, ^8^ The prospective study of Caramês et al. included Class 2 and 3 bone defects and provided the longest follow‐up (12 months) of all included studies. The horizontal bone gain at 12 months was 3.0 mm (2.5–3.5) at the implant neck and 3.5 mm (2.9–4.1) mid‐implant.

The review of the horizontal staged approach analyzed five clinical trials (three RCTs and two case series). In the study by Eskan et al.^55^ (RCT comparing biomaterial + collagen membrane vs. biomaterial + APC + collagen membrane), the APC group obtained significantly more bone gain. However, the results were contradictory in the other two RCTs, which compared using a collagen membrane or not. The results in the study by Aboelela et al.^57^ were better when a collagen membrane was used as a barrier versus a CGF membrane. These findings could result from the fast resorption of the CGF membrane. Conversely, the study by Tony et al.,^58^ where sticky bone was used with and without a collagen membrane, found better results in the group without the membrane. These two RCTs reflect the controversial data for the use of APC membranes as the sole barrier. On the other hand, the two case series^56^, ^59^ obtained a horizontal bone increase of 4.6 and 5.9 mm, respectively. These data are superior to what is described in the literature for horizontal bone augmentation with a staged approach.^7^, ^15^

The review of the vertical staged approach only identified two clinical studies (one RCT and one retrospective case series study). In the RCT, the APC group obtained statistically significantly better results than the non‐APC group. In the retrospective study, the amount of vertical gain was 5.6 mm, very similar to other techniques.^18^, ^20^, ^21^

In addition to these clinical data, several in‐vitro studies have demonstrated the molecular beneficial effects of APCs on osseous cell linage: increase of the BMP‐2 production,^64^ inhibition of osteoclastogenesis,^65^ shifts of the macrophage polarization from M1 to M2 phenotype,^66^ and increase of metabolic activity (alkaline phosphatase activity) and proliferation of human osteoblasts.^67^

There are no clinical studies in the literature comparing APCs with other biological molecules, such as enamel matrix proteins (EMD) or rhPDGF‐BB, in bone augmentation. However, a preclinical in‐vivo study on oral mucosal wound healing found no differences between combining collagen membranes with PRF, PRP, or EMD.^68^ Moreover, a randomized clinical trial comparing PRF membrane versus collagen membrane plus rhPDGF‐BB in localized intrabony defects found no differences between groups.^69^

### 4.1 Limitations and future research

Even though animal trials indicated some significant adjunct effects when APCs in a flowable form to create sticky bone were added to a bone substitute (particulate or block) during horizontal/vertical bone augmentation, the number of RCTs reporting it is rather small. Only three RCTs compare the standard of care with the new technique (sticky bone). In addition, no RCTs were found on the use of an L‐PRF bone block, probably because this approach is still quite new. Thus, strong recommendations cannot be made.

Another limitation is that the APC protocols are heterogeneous, which makes comparisons between studies difficult. Similarly, the studies' different follow‐up periods do not allow comparisons.

Furthermore, The individual phenotype should also be considered a variable since it will influence the final bone augmentation. Quirynen et al.^11^ examined eight patients with 15 GBRs (applying several strategies). They followed the long‐term dimensional changes of the graft on consecutive CBCTs (pre‐GBR, immediately post‐GBR, ≥ 6 months post‐GBR, and at least ≥24 months post‐GBR). These dimensional changes (graft resorption) were compared to the bone dimensions of the intact contra‐lateral alveolar bone, which represented the individual genotypical dimension (IGD) of the alveolar crest. The contra‐lateral site was laid (mirrored) over the augmented site and registered accordingly. Thus, early and late resorption could be compared to the individual genotypically dimensions (per millimeter apically from the alveolar crest, in the center of the GBR) as well as in 3D (the entire GBR, 2 mm away from the mesial, distal, and apical borders for standardization). After early resorption, the outline of the GBR was generally located ±1 mm outside the IGD, but after late resorption (±24 months after GBR, ±18 months after re‐entry for implant insertion), it moved further toward the IGD, with 88% of the measurements within a 0.5‐mm distance from the IGD. More detailed analyses also indicated no differences between the gold‐standard approach (50% particulate bone/50% xenograft + collagen membrane) and the use of an L‐PRF bone block.

Another important aspect is the use of L‐PRF membranes as a functional barrier covering a bony graft during horizontal/vertical bone regeneration. Occlusive barrier membranes play an important role during GBR since they prevent the down‐growth of the rapidly growing soft tissues and keep a space for bone regeneration. However, at the same time, they isolate the periosteum, which is considered the main source of progenitor cells and osteogenic mediators. Several studies examined whether L‐PRF membranes (with their release of growth factors, matrix proteins, and antibacterial capacity, among others) could be a useful alternative. Lee et al.^70^ created mesially and distally contained lateral ridge defects in the mandible of eight mongrel dogs and applied six different protocols via a split‐mouth concept: deproteinized bovine bone mineral (DBBM) or β‐tricalcium phosphate as bone substitute covered with either a non‐resorbable membrane, a resorbable membrane, or an L‐PRF membrane. The L‐PRF + DBBM group showed significantly more newly formed bone in histology and micro‐CT analyses. In turn, Park et al.^54^ prepared similar defects in beagle dogs and treated them with DPBM and a collagen membrane, DPBM mixed with i‐PRF, or the latter covered with an L‐PRF membrane. They did not observe differences in the bone formation patterns between the three groups.

Hartlev et al.^71^, ^72^, ^73^ compared the outcome of a horizontal bone block augmentation (ramus graft + bone particles) covered by either an L‐PRF membrane alone or by deproteinized bovine bone mineral plus a resorbable collagen membrane (the gold standard). CBCT was taken before grafting and 2 weeks and 6 months after grafting. The volumetric changes between these 3 CBCTs were evaluated by planimetric measurements on two‐dimensional CBCT cross‐sections of the grafted regions,^73^ and a similar bone volumetric loss was observed (14.7% ± 8.9% in the L‐PRF group vs. 17.8% ± 13.3%). Histomorphometric analyses of the augmented bone 6 months after grafting revealed no significant differences between the groups: a mean of 14% vital bone, 80% non‐vital bone, 5% soft tissue, and 1% blood vessels in the L‐PRF group versus 14% vital bone, 63% non‐vital bone, 22% soft tissue, and 1% blood vessels in the gold‐standard group.^71^ At the final follow‐up visit (14–32 months after crown placement), both groups presented similar clinical peri‐implant parameters but with less marginal bone loss in the L‐PRF group.

These data agree with the observations from Rexhepi et al.,^74^ who compared the outcome of an L‐PRF membrane with a collagen membrane as a barrier membrane after applying an inorganic bone graft in unfavorable infra‐bony defects. Twelve months after surgery, clinical and radiographic parameters had improved significantly at both experimental sites. The L‐PRF group showed 0.5 mm more probing depth reduction, 0.8 mm more CAL gain, and 0.6 mm more bone gain.

More RCTs are definitely required to confirm the preliminary data on the benefits of applying APCs (flowable and/or solid, sticky bone vs. L‐PRF block) to further improve the outcome of GBR. Namely, comparisons with the gold‐standard approaches using resorbable or non‐resorbable membranes would be very useful.

## 5 CONCLUSION

The current data on the benefits of APCs in bone augmentation are scarce. However, most studies, particularly comparative and well‐designed studies, demonstrated benefits and promising results. Further randomized clinical studies are necessary to better understand the regenerative potential of APCs in horizontal and vertical bone augmentation and to test the efficacy of these platelet concentrates.

## Data Availability

Data sharing not applicable to this article as no datasets were generated or analysed during the current study.
